# PBF509, an Adenosine A_2A_ Receptor Antagonist With Efficacy in Rodent Models of Movement Disorders

**DOI:** 10.3389/fphar.2018.01200

**Published:** 2018-10-19

**Authors:** Fabiana Núñez, Jaume Taura, Juan Camacho, Marc López-Cano, Víctor Fernández-Dueñas, Naomi Castro, Julio Castro, Francisco Ciruela

**Affiliations:** ^1^Unitat de Farmacologia, Departament Patologia i Terapèutica Experimental, Facultat de Medicina, IDIBELL, Universitat de Barcelona, L’Hospitalet de Llobregat, Barcelona, Spain; ^2^Institut de Neurociències, Universitat de Barcelona, Barcelona, Spain; ^3^PaloBiofarma S.L., Barcelona, Spain

**Keywords:** PBF509, Parkinson’s disease, adenosine A_2A_ receptor, catalepsy, label-free, tremor, hemiparkinsonism, antagonist

## Abstract

Adenosine A_2A_ receptor (A_2A_R) antagonists have emerged as complementary non-dopaminergic drugs to alleviate Parkinson’s disease (PD) symptomatology. Here, we characterize a novel non-xhantine non-furan A_2A_R antagonist, PBF509, as a potential pro-dopaminergic drug for PD management. First, PBF509 was shown to be a highly potent ligand at the human A_2A_R, since it antagonized A_2A_R agonist-mediated cAMP accumulation and impedance responses with K_B_ values of 72.8 ± 17.4 and 8.2 ± 4.2 nM, respectively. Notably, these results validated our new A_2A_R-based label-free assay as a robust and sensitive approach to characterize A_2A_R ligands. Next, we evaluated the efficacy of PBF509 reversing motor impairments in several rat models of movement disorders, including catalepsy, tremor, and hemiparkinsonism. Thus, PBF509 (orally) antagonized haloperidol-mediated catalepsy, reduced pilocarpine-induced tremulous jaw movements and potentiated the number of contralateral rotations induced by L-3,4-dihydroxyphenylalanine (L-DOPA) in unilaterally 6-OHDA-lesioned rats. Moreover, PBF509 (3 mg/kg) inhibited L-DOPA-induced dyskinesia (LID), showing not only its efficacy on reversing parkinsonian motor impairments but also acting as antidyskinetic agent. Overall, here we describe a new orally selective A_2A_R antagonist with potential utility for PD treatment, and for some of the side effects associated to the current pharmacotherapy (i.e., dyskinesia).

## Introduction

Parkinson’s disease (PD) is a neurodegenerative condition affecting around 1% of the population over the age of 65 (Meissner et al., 2011). PD is characterized by bradykinesia, tremor, and rigidity, which are secondary to the loss of dopamine neurons in the substantia nigra (Poewe and Mahlknecht, 2009). The main therapeutic approach consists of administrating L-3,4-dihydroxyphenylalanine (L-DOPA) or dopamine receptor agonists, thus recovering the functioning of dopaminergic transmission (Poewe, 2009). However, a number of adverse effects appear upon the long consumption of dopamine-like based drugs (Huot et al., 2013). From these, dyskinesia is the most reported one, since, in most cases, it critically impedes the normal life of patients. Rotation of dopamine-like based drugs is a normal strategy to diminish the appearance of these secondary effects, but it seems clear that there is a need in PD clinics for searching novel agents that may improve the management of the pathology (Schapira et al., 2006).

From the new drugs developed not only to improve the clinical features of classical drugs but also to alleviate their undesired side effects, adenosine A_2A_ receptor (A_2A_R) antagonists appear to be the most promising ones. Interestingly, A_2A_Rs are expressed in GABAergic enkephalinergic neurons together with dopamine D_2_ receptors (D_2_R), which are the main target of PD drugs (Gerfen et al., 1990). In addition, it has been largely studied the presence of reciprocal functional interactions between both receptors, a phenomenon that has been postulated to permit a fine-tuning modulation of the basal ganglia functioning (for review see Ferre et al., 2004). Furthermore, it was recently demonstrated that both receptors form receptor complexes (i.e., oligomers), in which a direct receptor-receptor interaction may drive the functional interplay between A_2A_R and D_2_R (Fernández-Dueñas et al., 2015). Nevertheless, apart from the plausible pre- and post-synaptic mechanism mediating its effects (Schiffmann et al., 2007), it has been clearly shown that A_2A_R antagonists show an antiparkinsonian efficacy, and that they may also be used to lessen undesired side effects of dopaminergic-like based drugs. In such way, the obtained pre-clinical information points to the use of A_2A_R antagonists as valuable agents for: (i) providing motor benefits by themselves, (ii) potentiating the benefit of dopamine agonists, or (iii) preventing the development of dopamine-like drugs induced dyskinesias (for review see Hauser and Schwarzschild, 2005; Jenner et al., 2009). In line with this, randomized clinical studies have been performed to assess the efficacy of these new A_2A_R-based drugs (for review see Vallano et al., 2011). Indeed, an A_2A_R antagonist (i.e., istradefylline) (Jenner, 2005) has been licensed in Japan (Nouriast^®^) as an adjuvant to L-DOPA treatment in order to reduce off-times produced by the dopaminergic drug (Mizuno et al., 2010; Kondo and Mizuno, 2015; Müller, 2015).

The development of new A_2A_R antagonists is consequently a main objective in PD therapeutics, since they may represent alternative or complementary drugs to deal with the symptomatology associated with the pathology. Importantly, it is crucial the information gained in pre-clinical studies, which may permit to properly screen the better candidates to be tested in randomized clinical studies. However, finding the optimal animal model is not a simple task, and it is usually mandatory to demonstrate the activity of any new drug in a variety of them. Here, we describe the effectiveness of PBF509, a novel selective and potent A_2A_R antagonist, in several rat animal models of movement impairment: (i) the pilocarpine-induced tremulous jaw movement (TJM), (ii) the hemiparkinsonian animal model, and (iii) the L-DOPA induced dyskinesia (LID). Importantly, we show the activity of this new antiparkinsonian drug in comparison with other well-known A_2A_R-based agents, aiming to prompt its future use in randomized clinical trials.

## Materials and Methods

### Drugs

PBF509 (Mediavilla-Varela et al., 2017), synthesized by PaloBiofarma, was dissolved in 0.5% methylcellulose for oral administration. All other compounds were obtained from external sources: 6-hydroxydopamine (6-OHDA), benserazide, pilocarpine, haloperidol (Sigma-Aldrich, St. Louis, MO, United States), 3,4-Dihydroxy-L-phenylalanine (L-DOPA; Abcam Biochemicals, Cambridge, United Kingdom), CGS21680, SCH442416, (Tocris Bioscience, Ellisville, MO, United States).

### Antibodies

The primary antibodies used were rabbit anti-TH polyclonal antibody (Millipore, Temecula, CA, United States), mouse anti-A_2A_R monoclonal antibody (Millipore) and rabbit anti-α-actinin polyclonal antibody (Santa Cruz Biotechnology, Santa Cruz, CA, United States). The secondary antibodies were horseradish peroxidase (HRP)-conjugated goat anti-rabbit and goat anti-mouse IgG (Pierce Biotechnology, Rockford, IL, United States), and Cy2-conjugated donkey anti-rabbit and Cy2-conjugated donkey anti-mouse antibodies (Jackson ImmunoResearch Laboratories).

### cAMP Accumulation Inhibition Assay

cAMP accumulation was measured using the LANCE *Ultra* cAMP kit (PerkinElmer, Waltham, MA, United States) (Taura et al., 2016). In brief, HEK-293 cells permanently expressing the A_2A_R^SNAP^ construct (Fernández-Dueñas et al., 2015) were incubated in the absence or presence of increasing concentrations of SCH442416 or PBF509 before stimulating the cells with CGS21260 (∼EC_80_) for 30 min at 22°C with adenosine deaminase (0.5 U/ml; Roche). Eu-cAMP tracer and U*Light*^TM^-anti-cAMP reagents were prepared and added to the sample according to the LANCE^®^ *Ultra* cAMP Kit instruction manual. 384-wells plate was incubated 1 h at 22°C in the dark and was then read on a POLARstar microplate reader (BMG LABTECH, Durham, NC, United States). Measurement at 620 and 665 nm were used to detect the TR-FRET signal and the concomitant cAMP levels were calculated following manufacturer’s instructions. Data were fitted by non-linear regression using GraphPad Prism 5 (GraphPad Software).

Concentration-response curves were carried out by assaying different ligand (i.e., PBF509 and SCH442416) concentrations ranging between 10 nM to 30 μM. Data was expressed as K_B_ by following the formula reported by Leff and Dougall (1993):

$${\text{K}}_{\text{B}}={\text{IC}}_{50}/{\left[2+{\left([\text{A}]/[{\text{A}}_{50}]\right)}^{\text{n}}\right]}^{\left(1/\text{n}\right)}-1$$

Where IC_50_ is the concentration of compound that inhibits CGS21680 effect by a 50%; [A] is the concentration of CGS21680 employed in the assay, [A_50_] is the CGS21680 EC_50_ value and n is the Hill slope of the curve.

### Cellular Impedance Assay Label-Free

The xCELLigence RTCA system (Roche) was employed to measure changes in cellular impedance correlating with cell spreading and tightness, thus being widely accepted as morphological and functional biosensor of cell status (Xu et al., 2016). Accordingly, we assessed the impact of A_2A_R blockade in cellular impedance (Solly et al., 2004; Stallaert et al., 2012; Hillger et al., 2015). To this end, HEK-293 cells permanently expressing the A_2A_R^SNAP^ construct (Fernández-Dueñas et al., 2015) were growth in Dulbecco’s modified Eagle’s medium (DMEM) (Sigma-Aldrich) supplemented with 1 mM sodium pyruvate (Biowest, Nuaillé, France), 2 mM L-glutamine (Biowest), 100 U/mL streptomycin (Biowest), 100 mg/mL penicillin (Biowest), and 1.5% (v/v) fetal bovine serum (Gibco) in presence of 0.5 U/ml of adenosine deaminase. The 16-wells E-plates (Roche) were used. Wells were coated with 50 μl fibronectin (10 μg/ml). Plates were placed at 37°C for 1 h. After removing coating liquid, plates were washed three times with 100 μl Milli-Q-water before use. The background index for each well was determined with supplemented DMEM (90 μl) in the absence of cells. Data from each well were normalized to the time point just before compound addition using the RTCA software providing the normalized cell index (NCI). Subsequently, cells (90 μl re-suspended in supplemented DMEM were then plated at a cell density of 10,000 cells/well and grown for 18 h in the RTCA SP device station (Roche) at 37°C and in an atmosphere of 5% CO_2_ before ligand (i.e., CGS21680) addition. For the concentration-response inhibition of CGS21680-mediated effect the cells were first incubated with the corresponding antagonist (i.e., PBF509 or SCH442416) for 1 h and then CGS21680 (∼EC_80_) was added. Cell index values were obtained immediately following ligand stimulation every 15 s for a total time of at least 100 min. For data analysis, ligand (i.e., SCH442416 and PBF509) responses were transformed to ΔNCI after subtracting baseline (i.e., vehicle control) to correct for any ligand-independent effects. The ΔNCI was then taken at 30 min after the agonist addition to build the concentration-response curve. ΔNCI were expressed in % considering 1 μM of CGS21680 as the 100% and the vehicle as the 0%.

### Animals

Sprague-Dawley rats (Charles River Laboratories, L’Arbresle, France) weighing 240–250 g were used. The University of Barcelona Committee on Animal Use and Care approved the protocol. Animals were housed and tested in compliance with the guidelines provided by the Guide for the Care and Use of Laboratory Animals (Clark et al., 1997) and following the European Union directives (2010/63/EU). All efforts were made to minimize animal suffering and the number of animals used. Rats were housed in groups of three in standard cages with *ad libitum* access to food and water and maintained under a 12 h dark/light cycle (starting at 7:30 AM), 22°C temperature, and 66% humidity (standard conditions). All animal model observations were made between 9:00 AM and 1:00 PM.

### Haloperidol-Induced Catalepsy

Rats (*n* = 10) were randomly assigned to treatment groups and behavioral testing was performed blind to treatment. The dopamine D_2_ receptor (D_2_R) antagonist, haloperidol (1 mg/kg, s.c.) was administered to induce catalepsy. Thirty minutes after the haloperidol administration, rats experienced a full cataleptic response. At this time point, for each rat the state of catalepsy was tested by gently placing their front limbs over an 8-cm high horizontal bar. The intensity of catalepsy was assessed by measuring the time the rats remain in this position being completely immobile for a maximum of 120 s. Only rats that remained cataleptic for the entire 120 s were used for subsequent drug testing. After 30 min of the baseline measurement vehicle (0.5% methylcellulose and 2% DMSO) or PBF509 was administered orally via gavage (3, 10, or 30 mg/kg, p.o.) and the catalepsy was then determined at 15, 30, and 60 min PBF509 administration. For each time point the number of responding rats and the total cataleptic time for each animal was determined.

### Pilocarpine-Induced TJM

Rats were placed in the observation chamber (30 cm diameter and 40 cm high clear glass chamber with a mesh floor and elevated 40 cm from the bench) to habituate during 5 min before being orally administered with vehicle (0.5% methylcellulose and 2% DMSO) or the indicated A_2A_R antagonist (i.e., SCH442416 and PBF509), followed (20 min) by pilocarpine (1 mg/kg; i.p.). Five minutes after pilocarpine injection TJMs were counted for 1 h (divided into six tests of 10 min each). TJMs were defined as rapid vertical deflections of the lower jaw that resembled chewing but were not directed at any particular stimulus (Salamone et al., 1998). Each individual deflection of the jaw was recorded using a mechanical hand counter by a trained observer, who was blind to the experimental condition of the rat being observed (Gandía et al., 2015).

### Hemiparkinsonian Animal Model

Experimental hemiparkinsonism was induced in rats by unilateral injection of 6-OHDA in the medial forebrain bundle as previously described (Fernández-Dueñas et al., 2015). Accordingly, rats were stereotaxically injected with 6-OHDA (8 μg of 6-OHDA in 4 μl of saline solution containing 0.05% ascorbic acid) at: AP (anterior-posterior) = −2.2 mm, ML (medial-lateral) = −1.5 mm and DV (dorsal-ventral) = −7.8 mm with respect to bregma (Paxinos and Watson, 2007). To minimize damage to noradrenergic neurons, rats were pretreated with desipramine hydrochloride (10 mg/kg, i.p.) 20 min before surgery. Then, 3 weeks after the lesion the extent of dopamine deafferentation was validated by assessing the rotating behavioral response to L-DOPA administration. In brief, rats were injected with L-DOPA (50 mg/kg, i.p.) in the presence of benserazide hydrochloride (25 mg/kg, i.p.) and the number of full contralateral turns recorded during a 2 h period. Dopamine deafferentation was considered successful in those animals that made at least 200 net contralateral rotations. Thereafter, animals were housed during 3 weeks before used.

To test the effect of A_2A_R antagonists in the hemiparkinsonian animal model compounds were orally administered in vehicle (0.5% methylcellulose and 2% DMSO) 40 min before the administration of benserazide (25 mg/kg; i.p.). Subsequently, L-DOPA (4 mg/kg; i.p.) was delivered 20 min later and placed in the rotametry chambers, as previously described (Hodgson et al., 2009). The number of contralateral rotations was recorded during a 2-h period.

### LIDs and Abnormal Involuntary Movements (AIMs) Rating

L-DOPA-induced dyskinesias were triggered to the hemiparkinsonian rats (see above) by twice a day administration of L-DOPA (4 mg/kg, i.p.) plus benserazide hydrochloride (15 mg/kg, i.p.) during 22 consecutive days. Subsequently, the L-DOPA-induced AIMs were scored by a blinded experimenter following a rat dyskinesia scale previously described (Winkler et al., 2002). In brief, rats were injected with L-DOPA, placed individually in transparent plastic cages and observed every 20 min during 220 min. Thus, three subtypes of AIMs were monitored (i.e., axial, forelimb, and orolingual) and their respective severity scored from 0 to 4 as previously described (Winkler et al., 2002). Enhanced manifestations of otherwise normal behaviors, such as rearing, sniffing, grooming, and gnawing, were not included in the rating. Accurate AIM ratings were subsequently performed on treatment days 1, 7, 14, and 22 during the chronic L-DOPA administration phase. We computed integrated AIM scores for each animal and testing session using the sum of all three AIM subtypes and expressed as the area under the curve (AUC).

### Gel Electrophoresis and Immunoblotting

Sodium dodecyl sulfate polyacrylamide gel electrophoresis (SDS/PAGE) was per-formed using 10% polyacrylamide gels. Proteins were transferred to PVDF membranes using a semidry transfer system and immunoblotted with the indicated antibody and then HRP-conjugated rabbit antigoat (1:30,000) or goat anti-rabbit IgG (1:30,000). The immunoreactive bands were developed using a chemiluminescent detection kit (Pierce) and the Amersham Imager 600 (GE Healthcare Europe GmbH, Barcelona, Spain).

### Immunohistochemistry

Rat brains were fixed and coronal sections (50–70 μm) obtained as previously described (Taura et al., 2015). Slices were collected in Walter’s Antifreezing solution (30% glycerol, 30% ethylene glycol in PBS, pH 7.2) and kept at −20°C until processing.

For immunohistochemistry, previously collected slices were washed three times in PBS, permeabilized with 0.3% Triton X-100 in PBS for 2 h and rinsed back three times more with wash solution (0.05% Triton X-100 in PBS). The slices were then incubated with blocking solution (10% NDS in wash solution; Jackson ImmunoResearch Laboratories, Inc., West Grove, PA, United States) for 2 h at R.T. and subsequently incubated with rabbit anti-TH polyclonal antibody (1 μg/ml) and mouse anti-A_2A_R monoclonal antibody (1 μg/ml) overnight at 4°C. After two rinses (10 min each) with 1% NDS in wash solution, sections were incubated for 2 h at R.T. with either Cy2-conjugated donkey anti-rabbit (1:200) or Cy2-conjugated donkey anti-mouse (1:200) antibodies before being washed (10 min each) two times with 1% NDS in wash solution and two more times with PBS and mounted on slides. Fluorescence images of whole brain coronal sections were obtained using a SteREO Lumar.V12 fluorescence stereoscope (Carl Zeiss MicroImaging GmbH, Oberkochen, Germany).

### Statistics

The number of samples (n) in each experimental condition is indicated in figure legends. When two experimental conditions were compared, statistical analysis was performed using an unpaired *t*-test. Otherwise, statistical analysis was performed by one-way analysis of variance (ANOVA) followed by Bonferroni *post hoc* test. Statistical significance was set as *P* < 0.05.

## Results

### Functional Activity of PBF509 at Human Recombinant A_2A_R

PBF509 is a structurally novel non-xanthine and non-furan A_2A_R antagonist. The affinity of PBF509 for all four human adenosine receptors was recently reported by means of classical radioligand competition binding assays using membrane extracts from cells expressing A_1_R, A_2A_R, A_2*B*_R, and A_3_R (Mediavilla-Varela et al., 2017). Thus, PBF509 bound A_2A_R with high affinity (*K*_i_ = 12 ± 0.2 nM) and showed 416-, 208-, and 83-fold selectivity over the A_3_R, A_1_R, and A_2*B*_R, respectively (Mediavilla-Varela et al., 2017).

**FIGURE 1:**
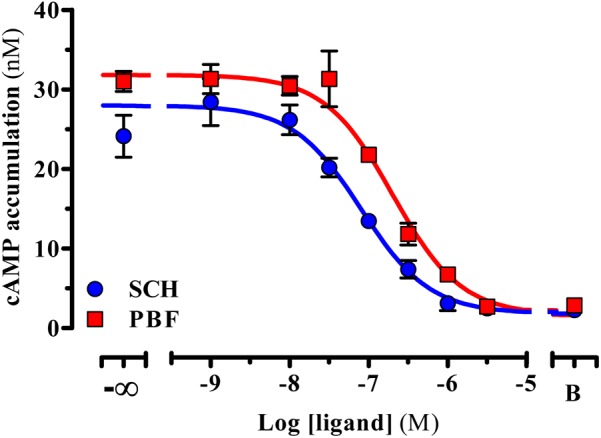
Inhibition of A_2A_R-mediated cAMP accumulation. Inhibition of the CGS21680-mediated cAMP accumulation. A SCH442416 and PBF509 concentration-response inhibition curve of CGS21260-mediated cAMP was assessed in HEK-293 cells permanently expressing the A_2A_R^SNAP^. Data are representative of three separate experiments performed in duplicate.

In functional assays, PBF509 did not show any agonist efficacy in HEK cells permanently expressing the human A_2A_R^SNAP^ (data not shown). However, PBF509 completely antagonized the agonist-mediated cAMP accumulation in A_2A_R^SNAP^ expressing HEK cells (**Figure 1**), thus showing a K_B_ value of 72.8 ± 17.4 nM (**Figure 1**). Interestingly, while the PBF509 K_B_ value was significantly different [*F*_(1,60)_ = 11.5, *P* < 0.005] from the one found for a well characterized A_2A_R antagonist (Todde et al., 2000), SCH442416 (*K*_B_ = 28.8 ± 7.2 nM; **Figure 1**), it was within the same range as previously described (Mediavilla-Varela et al., 2017). Thus, these results suggested that PBF509 was equipotent in blocking A_2A_R-mediated cAMP accumulation at the moderate nanomolar range.

**FIGURE 2:**
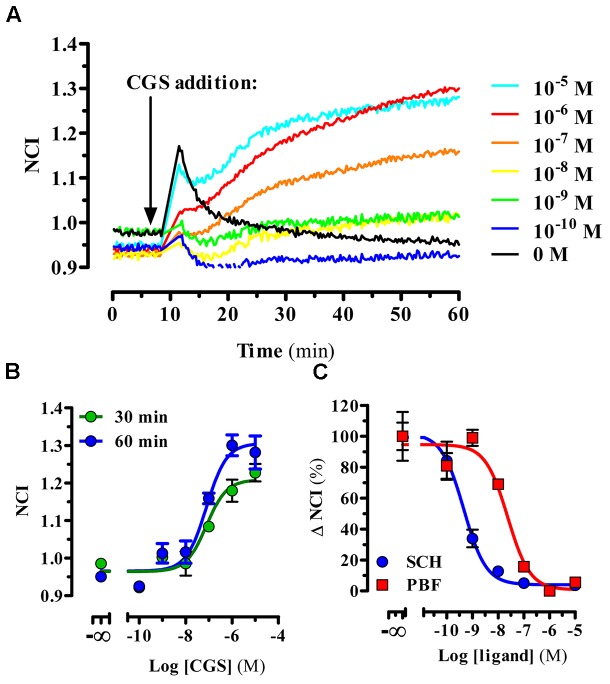
PBF509 blocks A_2A_R-mediated whole cell label-free responses. **(A)** Representative example of the A_2A_R^SNAP^ cells impedance signal in response to CGS216980 (10 μM–100 pM) over the time. Cell lines were stimulated with the A_2A_R selective agonist CGS21680 18 h after seeding (10,000 cells/well) and the impedance signal recorded over 60 min as described in materials and methods. **(B)** Concentration-response curves of CGS21680 derived from the normalized cell index (NCI) shown in **(A)** within 30 or 60 min after agonist addition. EC_50_ values of CGS21680 were 127 ± 74 and 61 ± 31 nM at 30 and 60 min, respectively. **(C)** Inhibition of the CGS21680-mediated impedance signal. Cell lines were pre-incubated for 60 min with increasing concentrations of SCH442416 and PBF509 (10 μM–100 pM) before stimulation with CGS21680 (500 nM). Concentration-response curves of SCH442416 and PBF509 were derived from ΔNCI within 30 min after agonist addition. Data are representative of three separate experiments performed in duplicate.

Next, we aimed to characterize the functional activity of PBF509 using a label-free technology. To this end, the whole-cell agonist-mediated impedance responses were monitored in the presence or absence of PBF509 using a biosensor method. Once completed the optimization of the assay (see section “Materials and Methods”), we first tested the CGS21680-mediated changes in morphology (i.e., impedance) of A_2A_R^SNAP^ expressing HEK cells, which were recorded in real-time. Interestingly, addition of CGS21680 resulted in an immediate and concentration-dependent increase of impedance (**Figure 2A**). The EC_50_ found for this CGS21680-mediated impedance change was of 127 ± 74 and 61 ± 31 nM, for measurements performed at 30 and 60 min, respectively (**Figure 2B**). As the potency value did not significantly differ between the two time points measured [*F*_(1,21)_ = 1.256, *P* = 0.274], we assessed the ability of PBF509 to block the CGS-induced impedance change at 30 min (**Figure 2C**). Of note, while SCH442416 showed a K_B_ value of 0.2 ± 0.07 nM, PBF509 displayed a K_B_ of 8.2 ± 4.2 nM (**Figure 2C**). Thus, the PBF509 inhibitory potency of A_2A_R-mediated impedance increase was within the low nanomolar range, following the same tendency to that displayed by SCH442416. Overall, the two functional assays used confirmed a lower potency of PBF509 vs. SCH442416 in blocking the A_2A_R-mediated signaling, which ranged from 2.5-fold in the cAMP assay and 40-fold in the label-free approach. Importantly, the potency values derived from the label-free assay provided a proof-of-principle that this biosensor technology can be applied to pharmacologically characterize A_2A_R-based drugs.

### PBF509 Attenuates Haloperidol-Induced Catalepsy

**FIGURE 3:**
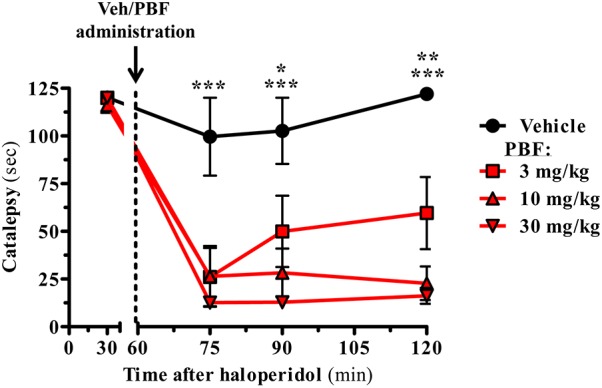
PBF509 reverses haloperidol-induced catalepsy. Rats were pretreated with haloperidol (1.0 mg/kg, s.c.) and 1 h later the selected cataleptic animals were orally administered with either vehicle or PBF509 (3, 10, and 30 mg/kg, p.o.). The time spent in a cataleptic position was measured after 15, 30, and 60 min after PBF509 administration. The data represent the mean time spent cataleptic ± SEM over a period of 120 s measurement (*n* = 3–8 animals/group). The cataleptic behavior was calculated and compared within groups by a two-way ANOVA followed by Bonferroni’s *post hoc* test. Group differences were calculated by a non-parametric Kruskal Wallis ANOVA followed by a Dunn’s post-test as Gaussian distribution was missing. ^∗^*P* < 0.05, ^∗∗^*P* < 0.01, ^∗∗∗^*P* < 0.001. The alpha-error level was set to 0.05.

Once determined the potency and intrinsic activity of PBF509 we aimed to determine the pro-dopaminergic nature of this compound *in vivo*. To this end, we evaluated the ability of PBF509 to block D_2_R antagonist-mediated catalepsy. PBF509 dose-dependently attenuated the cataleptic effects of haloperidol when administered 1 h after haloperidol injection (**Figure 3**). Interestingly, the anticataleptic activity of PBF509 was observed after 15 min of administration at all the doses tested (3, 10, and 30 mg/kg) and lasted for at least 1 h post-administration of PBF509 (**Figure 3**). These results are in good agreement with the early description of anticataleptic activity of A_2A_R antagonists (Kanda et al., 1994).

### PBF509 Attenuates Pilocarpine-Induced Tremulous Jaw Movements

**FIGURE 4:**
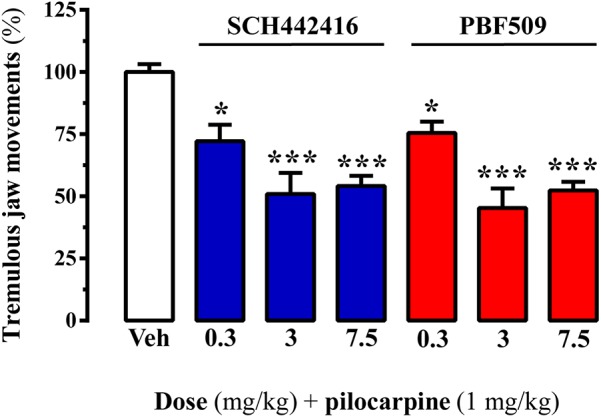
PBF509 attenuates pilocarpine-induced tremulous jaw movements. Effect of different doses of SCH442416 and PBF509 on pilocarpine-induced tremulous jaw movements. The number of jaw movements were recorded during 1 h in rats orally administered with vehicle (Veh), SCH442416 or PBF509 (0.3–7.5 mg/kg) before (20 min) pilocarpine administration (1 mg/kg, i.p.). Values correspond to the mean ± SEM of 6–7 animals for each condition and expressed as percentage of the TJMs observed in the vehicle. TJMs were absent in animals no treated with pilocarpine. The asterisks denote data significantly different from the vehicle condition: ^∗^*P* < 0.05 and ^∗∗∗^*P* < 0.001, one-way ANOVA with a Bonferroni’s *post hoc* test.

The adenosinergic system has emerged as a potential target for the treatment of parkinsonian symptoms, including tremor (Schwarzschild et al., 2006). Indeed, A_2A_R antagonists have been shown to be efficacious to reduce drug-induced tremor (Simola et al., 2004). Accordingly, we aimed to test whether PBF509 was able to reduce pilocarpine-induced tremulous jaw movements (TJMs), an animal model of parkinsonian tremor previously used (Salamone et al., 2013; Gandía et al., 2015). Interestingly, PBF509 dose-dependently attenuated pilocarpine-induced TJMs, being effective at the lowest dose tested (0.3 mg/kg; **Figure 4**). It is important to mention here that PBF509 showed similar potency to SCH442416 in reducing TJMs (**Figure 4**). Thus, these results suggested that parkinsonian rest tremor, which is relatively resistant to dopamine-replacement therapy, might be potentially targeted by PBF509.

### Effect of PBF509 on L-DOPA-Induced Turning Behavior

**FIGURE 5:**
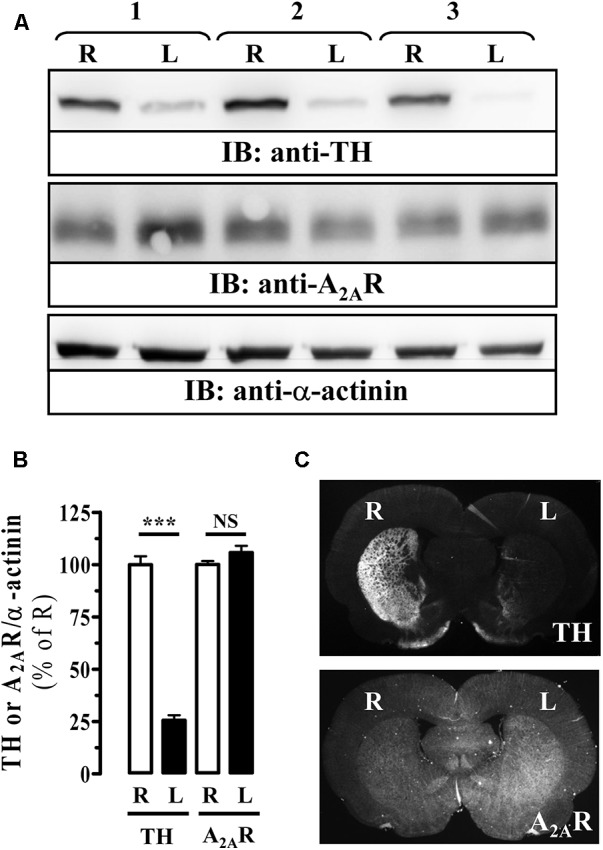
Immunoreactivity of A_2A_R in the striatum of 6-OHDA-lesioned rats. **(A)** Immunoblot analysis of TH and A_2A_R density in striatal membranes from control (R) and 6-OHDA lesioned (L) striatal hemisphere. Striatal membranes were analyzed by SDS–PAGE and immunoblotted using a rabbit anti-TH polyclonal antibody (1 μg/ml), a mouse anti-A_2A_R monoclonal antibody (1 μg/ml) and a rabbit anti-α-actinin polyclonal antibody (1 μg/ml). A HRP-conjugated anti-rabbit or anti-mouse IgG (1/30,000) was used as a secondary antibody. The immunoreactive bands were visualized by chemiluminescence. Load control used for quantitating was α-actinin. A representative blot for three different lesioned animals is shown. **(B)** Quantification of TH and A_2A_R density in striatal membranes from control (R) and 6-OHDA lesioned (L) striatal hemisphere. The immunoreactive bands corresponding to TH and A_2A_R in each striatal hemisphere were normalized by α-actinin immunoreactivity. Data are expressed as percentage of the control (R) TH or A_2A_R density ± SEM of three independent experiments. Asterisks indicate data significantly different from the control condition: ^∗∗∗^*P* < 0.0001 by Student’s *t*-test. NS: not statistically significant. **(C)** Photomicrographs showing, by means of TH staining (upper panel), the loss of dopaminergic innervation in the lesioned striatum (L) compared to the non-lesioned (R) striatum, and the density of A_2A_R (lower panel) in 6-OHDA-lesioned rat brain coronal slices (see section “Materials and Methods”).

Next, we aimed to evaluate the antiparkinsonian effectiveness of PBF509 using the unilateral 6-OHDA lesioned rat, a classic and widespread toxin-based animal model of experimental parkinsonism (Schwarting and Huston, 1996). To this end, we first validated the extent of the 6-OHDA lesion in our hemiparkinsonian animal model by monitoring the striatal tyrosine hydroxylase (TH) density, a marker of dopaminergic innervation. As expected, a significant reduction (∼75%) of TH density in the lesioned striatum (L) was observed by immunoblot (**Figures 5A,B**) and immunohistochemistry (**Figure 5C**), thus corroborating the 6-OHDA-mediated loss of striatal dopaminergic innervation. Interestingly, when the A_2A_R immunoreactivity was assessed not significant differences (*P* = 0.193) between the healthy (R) and the lesioned (L) striatum were observed (**Figure 5**).

**FIGURE 6:**
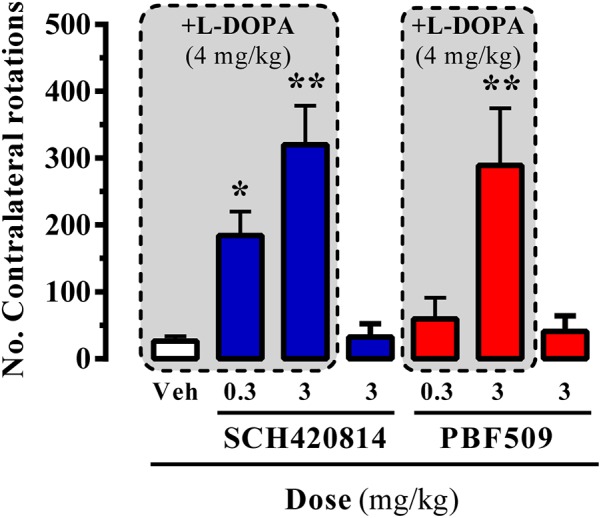
PBF509-mediated potentiation of L-DOPA-induced contralateral rotations in 6-OHDA-lesioned rats. The number of contralateral rotations in 6-OHDA-lesioned rats orally administered with vehicle (Veh) or SCH420814 and PBF509 (0.3 and 3 mg/kg) in the absence or presence of L-DOPA (4 mg/kg) was monitored during a 2 h period. Values correspond to the mean ± SEM of 10–15 animals for each condition. The asterisks denote data significantly different from the vehicle condition: ^∗^*P* < 0.05 and ^∗∗∗^*P* < 0.001, one-way ANOVA with a Bonferroni’s *post hoc* test.

In the hemiparkinsonian animal model, an asymmetry in motor behavior is produced upon administration of dopaminergic agents (i.e., L-DOPA), a consequence of the unilateral dopamine depletion in the nigrostriatal pathway (Duty and Jenner, 2011). Accordingly, pro-dopaminergic compounds can

promote contralateral rotations of lesioned animals upon submaximal L-DOPA dosing (4 mg/kg, for our hemiparkinsonian rats). The administration of A_2A_R antagonists, either SCH420814 (preladenant) or PBF509, up to 3 mg/kg, to 6-OHDA lesioned rats did not result in asymmetric turning behavior (**Figure 6**). However, both compounds dose-dependently induced a contralateral turning behavior when administrated before the subthreshold dose of L-DOPA (**Figure 6**). Overall, PBF509 was able to enhance the effects of L-DOPA with a minimum efficacious dose (MED) of 3 mg/kg p.o., and equal in efficacy to SCH420814 at this dose.

### PBF509 Reduces L-DOPA-Induced Dyskinesia (LID)

**FIGURE 7:**
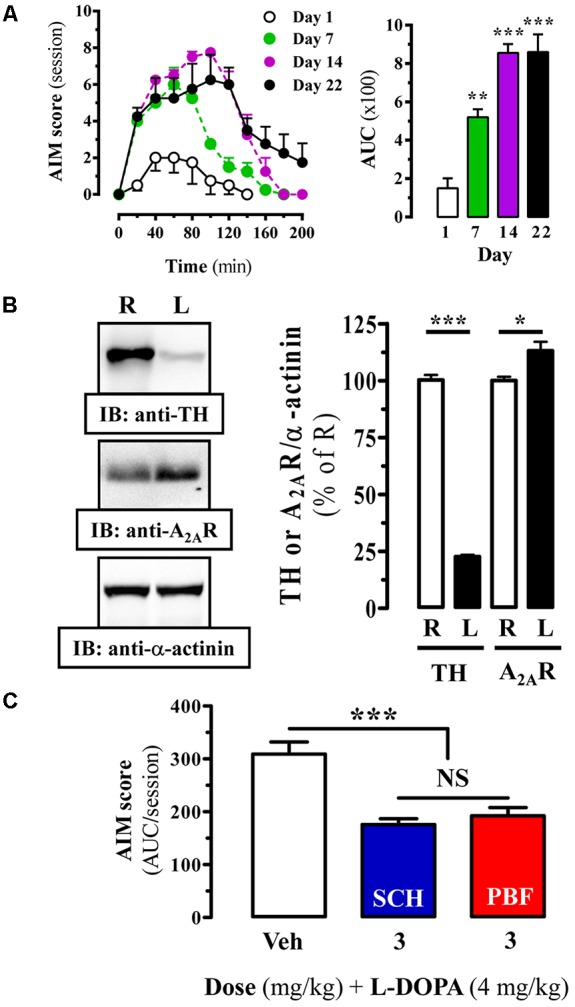
Effect of PBF509 in LID rats. **(A)** L-DOPA induced-motor side effects (i.e., LIDs) development upon chronic (22 days) L-DOPA (4 mg/kg) administration. AIM score was measured during a 220-min on days 1, 7, 14, and 22 after the corresponding daily L-DOPA administration. The total AIMs score over 200 min was quantified and expressed as area under the curve (AUC) ± SEM (*n* = 8). ^∗∗^*P* < 0.01 and ^∗∗∗^*P* < 0.001 one-way ANOVA with Dunnett’s *post hoc* test when compared to day 1. **(B)** Immunoreactivity of A_2A_R in the striatum of dyskinetic rats. TH and A_2A_R density in striatal membranes from control (R) and 6-OHDA lesioned (L) striatal hemisphere of LID animals was analyzed by immunoblot and quantified as described in **Figure 4**. Data are expressed as percentage of the control (R) TH or A_2A_R density ± SEM of three independent experiments. Asterisks indicate data significantly different from the control condition: ^∗∗∗^*P* < 0.001, ^∗^*P* < 0.05 by Student’s *t*-test. **(C)** LID attenuation in chronic (22 days) L-DOPA (4 mg/kg) administered rats following administration of A_2A_R antagonists. The total AIM score (area under the curve, AUC) obtained over 220 min following co-administration of L-DOPA (4 mg/kg) plus vehicle (Veh), SCH420814 (3 mg/kg) and PBF509 (3 mg/kg) are presented as mean score ± SEM (*n* = 6). The asterisks denote data significantly different from the vehicle condition: ^∗∗∗^*P* < 0.001, one-way ANOVA with a Bonferroni’s *post hoc* test. NS: not statistically significant.

Chronic L-DOPA use in PD is often associated with the development of LIDs. Accordingly, alleviating this adverse side effect related to PD therapy constitutes a therapeutic challenge. Interestingly, a relationship between A_2A_R and LIDs has been established, thus an increased striatal A_2A_R density has been reported in experimental animal models of LID (Zeng et al., 2000) and in PD patients with dyskinesia (Calon et al., 2004; Ramlackhansingh et al., 2011). Here, we aimed to assess the potential antidyskinetic activity of PBF509. To this end, we induced LIDs to our 6-OHDA lesioned rats by chronic L-DOPA administration and the emergence of abnormal involuntary movements (AIMs) over time was monitored. Indeed, a time L-DOPA-dependent AIMs manifestation was observed (**Figure 7A**). Thus, AIM severity increased during the first 40 min post-injection and remained significantly (*P* < 0.01) elevated for an additional 40 min in one-week L-DOPA administrated animals (**Figure 7A**, Day 7). A follow up of these animals over time showed that after 2 weeks of L-DOPA administration the AIMs remained robustly (*P* < 0.001) elevated during 80 min and after 3 weeks the animals showed a longer and sustained LIDs incidence (**Figure 7A**). Interestingly, the observed time-course in our LID animal model resembled the so called peak-dose dyskinesia in PD (Fahn, 2000). Subsequently, we next demonstrated that chronic L-DOPA treatment of hemiparkinsonian rats prompted a significant increase (15 ± 2%, *P* < 0.05) of striatal A_2A_R density in the lesioned hemisphere (**Figure 7B**), in agreement to that described previously. Then, under these experimental conditions, we assessed the antidyskinetic activity of PBF509. The drug was used at the antiparkinsonian MED (3 mg/kg, p.o.), and it showed an antidyskinetic efficacy similar to that observed for SCH420814 (**Figure 7C**). Overall, while PBF509 showed a robust antiparkinsonian activity it also displayed antidyskinetic efficacy.

## Discussion

Dopamine augmentation constitutes the first line therapy in PD. Hence, L-DOPA or direct dopamine agonists (i.e., ropinirole, pramipexole, apomorphine) are regular drugs for PD clinical management (Poewe and Mahlknecht, 2009). Interestingly, while dopamine-targeted therapies allow proper management of PD, associated motor disturbances have considerable side effects both after acute and chronic regimes (i.e., hallucinations, constipation, nausea, somnolence, on-off effects, dyskinesia) (Eggert et al., 2008). In addition, these therapies usually display an efficacy decline along the disease development and do not address other disease disturbances frequently associated to PD (i.e., mood, postural instability, or cognitive disturbances). Accordingly, alternative approaches modulating dopaminergic neurotransmission in PD have emerged as potential alternatives to manage PD therapy-associated side effects (Fox et al., 2008). Indeed, A_2A_R blockade has been demonstrated to be effective in both preclinical and clinical PD studies (Vallano et al., 2011; Pinna, 2014). Interestingly, it has been proposed that the mechanism behind the pro-dopaminergic activity of A_2A_R antagonists may rely in part to the existing functional and molecular interaction (i.e., heteromerization) of A_2A_R and D_2_R within the striatum (Ferre et al., 2004; Fernández-Dueñas et al., 2015). Thus, a mutual trans-inhibition between these two receptors has been largely described (Ferre et al., 2008). In addition to this postsynaptic site of action, striatal A_2A_R show also presynaptic expression on glutamatergic terminals of the cortico-limbic-striatal and thalamo-striatal pathways forming heteromeric complexes with adenosine A1 receptors (Ciruela et al., 2006) and driving striatal circuits controlling motor function independently of dopaminergic signaling (Schiffmann et al., 2007).

The initial work identifying novel A_2A_R antagonists focused on purine and xanthine derivatives, principally built from adenosine and the naturally occurring antagonist caffeine. In parallel, further effort was set on non-xanthine furan-based derivatives, such as triazolotriazines and triazolopyrimidines (e.g., ZM241385, BIIB014 and SCH420814). However, regardless of the good affinity and selectivity shown, these A_2A_R antagonists have, in general, high molecular weight, thus they are complex and difficult to synthesize. In addition, they display poor water solubility, and their furan group precludes replacement by empirical medicinal chemistry. Here, we characterize the functional activity of PBF509, a new non-xanthine and non-furan competitive antagonist with high affinity and selectivity for the A_2A_R (Mediavilla-Varela et al., 2017), through a highly sensitive TR-FRET-based cAMP accumulation assay in A_2A_R expressing cells. Interestingly, to further characterize the its activity we implemented a label-free xCELLigence assay based on the real-time impedance recording of A_2A_R expressing cells. Thus, upon similar physiological conditions of the xCELLigence assay (i.e., regular cell culture growing), PBF509 displayed comparable potency in blocking the A_2A_R-mediated signaling when compared to the cAMP assay. Noteworthy, apart from revealing PBF509 antiparkinsonian efficacy, our work led to the description of a whole-cell label-free approach for investigating A_2A_R-mediated drug responses in A_2A_R^SNAP^ expressing HEK cells, which may allow cellular assays with minimal modifications and increased sensitivity over traditional label-based methodologies.

PBF509 was effective in reducing catalepsy induced by haloperidol, a D_2_R antagonist. Interestingly, anticataleptic properties have been classically used to predict clinical efficacy for antiparkinsonian drugs (i.e., pramipexole) (Maj et al., 1997). Thus, our results suggested that PBF509-induced A_2A_R blockade provided a counterbalance to the loss of D_2_R-mediated effects in the basal ganglia indirect pathway, as previously described for other A_2A_R antagonists (Shiozaki et al., 1999). Moreover, PBF509-mediated A_2A_R blockade reduced pilocarpine-induced tremulous jaw movements and potentiated L-DOPA-induced contralateral rotations in unilaterally 6-OHDA-lesioned rats, which is consistent with previous findings using other A_2A_R antagonists (Hodgson et al., 2009). In addition, PBF509 showed antidyskinetic efficacy in the LID animal model, which correlated well with the increased striatal A_2A_R expression. Overall, our results support the potential usefulness of PBF509 in PD management, including its ability to reduce dyskinesia when used in combination with L-DOPA in PD treatment.

A number of A_2A_R antagonists have been proposed as antiparkinsonian agents and tested in preclinical PD animal models. For instance, ST-1535 (Rose et al., 2006; Tronci et al., 2007) and related metabolites (i.e., ST3932 and ST4206) (Stasi et al., 2015), and JNJ40255293 (Atack et al., 2014), which are based on the purine adenosine. Also, the xanthine-based compounds, as KW6002 (istradefylline) (LeWitt et al., 2008) and the non-xanthine SCH58261 (Simola et al., 2004), SCH420814 (preladenant) (Hodgson et al., 2009), and BIIB014 (vipadenant) (Gillespie et al., 2009), amongst others. However, among the A_2A_R antagonists undergoing clinical trials (Vallano et al., 2011; Pinna, 2014), only the xanthine istradefylline has been approved for manufacturing and marketing (in Japan, 2013), thus becoming the world’s first antiparkinsonian agent of a first-in-class A_2A_R antagonist. In clinical trials, Nouriast^®^ (istradefylline) improved wearing-off phenomena and was well tolerated by PD patients treated with L-DOPA. On the other hand, the non-xanthine preladenant did not prove itself to be more effective than placebo during Phase-III trials, and was discontinued in 2013, as it was vipadenant. Similarly, despite the robust preclinical pharmacology and good pharmacokinetic properties of JNJ40255293, its development was halted due to preclinical toxicity. Here, we have described a selective non-xanthine and non-furan A_2A_R antagonist with efficacy in rat models of movement disorders and without preclinical toxicity. Interestingly, in a double-blind, placebo-controlled, Phase-I clinical trial (NCT01691924) of single ascending oral doses in 32 healthy male volunteers, PBF509 showed safety, tolerability and feasibility. Thus, the compound is currently in prospective Phase-II clinical trials for PD.

In summary, PBF509 demonstrated remarkable potential in experimental animal models of movement disorders, including PD and LID, thus becoming an excellent candidate for clinical A_2A_R-based treatment of PD motor symptoms. In addition, the ability of PBF509 to alleviate non-motor symptoms associated with PD (i.e., memory and mood impairments and sleep disturbances) will deserve future clinical attention.

## Author Contributions

FN performed the *in vivo* experiments. JT performed the *in vitro* experiments. JaC synthesized the PBF509. ML-C performed the *in vivo* experiments and analyzed the data. VF-D designed the experiments and wrote the paper. NC conceived and supervised the project. JlC conceived and supervised the project and designed the experiments. FC conceived and supervised the project, designed the experiments, analyzed the data, and wrote the paper.

## Conflict of Interest Statement

The authors declare that the research was conducted in the absence of any commercial or financial relationships that could be construed as a potential conflict of interest. The reviewer RC declared a past co-authorship with the authors to the handling Editor.
